# Misperception of peer beliefs reinforces inequitable gender norms among Tanzanian men

**DOI:** 10.1017/ehs.2024.6

**Published:** 2024-02-21

**Authors:** David W. Lawson, Zhian Chen, Joseph A. Kilgallen, Charlotte O. Brand, Alexander M. Ishungisa, Susan B. Schaffnit, Yusufu Kumogola, Mark Urassa

**Affiliations:** 1 Department of Anthropology, University of California, Santa Barbara, USA; 2 Human Behaviour and Cultural Evolution Group, College of Life and Environmental Sciences, University of Exeter, Penryn, UK; 3 National Institute for Medical Research, Mwanza, Tanzania; 4 Muhimbili University of Health and Allied Sciences, Dar es Salaam, Tanzania; 5 Department of Anthropology, Pennsylvania State University, USA

**Keywords:** Cultural evolution, social learning, social norms, global health, gender

## Abstract

Gender role ideology, i.e. beliefs about how genders should behave, is shaped by social learning. Accordingly, if perceptions about the beliefs of others are inaccurate this may impact trajectories of cultural change. Consistent with this premise, recent studies report evidence of a tendency to overestimate peer support for inequitable gender norms, especially among men, and that correcting apparent ‘norm misperception’ promotes transitions to relatively egalitarian beliefs. However, supporting evidence largely relies on self-report measures vulnerable to social desirability bias. Consequently, observed patterns may reflect researcher measurement error rather than participant misperception. Addressing this shortcoming, we examine men's gender role ideology using both conventional self-reported and a novel wife-reported measure of men's beliefs in an urbanising community in Tanzania. We confirm that participants overestimate peer support for gender inequity. However, the latter measure, which we argue more accurately captures men's true beliefs, implies that this tendency is relatively modest in magnitude and scope. Overestimation was most pronounced among men holding relatively inequitable beliefs, consistent with misperception of peer beliefs reinforcing inequitable norms. Furthermore, older and poorly educated men overestimated peer support for gender inequity the most, suggesting that outdated and limited social information contribute to norm misperception in this context.

## Graphical abstract

**Figure:**
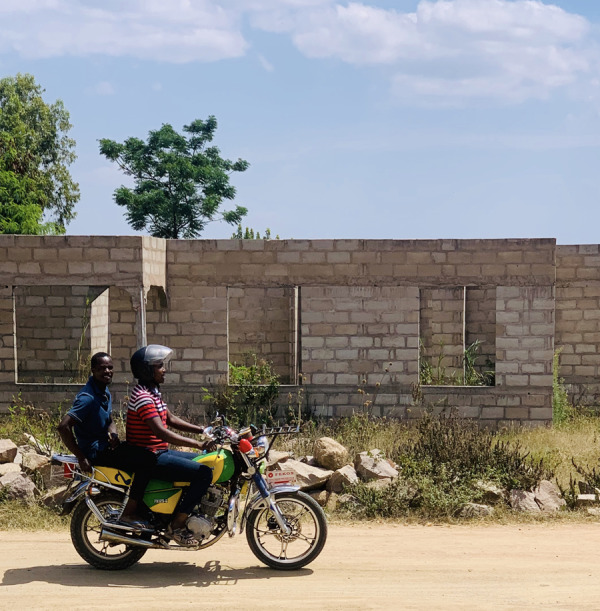

**Social media summary:** Misperception of peer beliefs reinforces inequitable gender norms among Tanzanian men.

## 1. Introduction

### 1.1. The social learning of gender role ideology

Gender role ideology can be defined as beliefs or attitudes individuals hold regarding the appropriate roles, rights and responsibilities of women and men in society (Krosta 2007). Gender role ideology differs markedly across and within cultures, ranging from emphasis on distinctive roles for each gender, typically favouring male privilege, to more egalitarian beliefs. Global health practitioners working to promote positive change in gender role ideology, including advances in women's empowerment, increasingly emphasise the importance of targeting gender norms, i.e. shared standards of appropriate behaviour for each gender (Jayachandran, 2015; Stewart et al., 2021). From this perspective, individual gender role ideology may vary within a group, but is nevertheless strongly influenced by perceptions of what others do and think, and the anticipated rewards or punishments for conforming to or deviating from perceived norms (Berkowitz et al., 2022; Bicchieri, 2017).

This ‘social norms approach’ complements studies of cultural evolution, which also address the role of social information, conformity and normative sanctions in guiding behavioural variation (Kendal et al., 2018; Mesoudi, 2011). However, to our knowledge, scholars of cultural evolution have rarely addressed gender role ideology (but see Cross et al., 2023; O'Connor, 2019), or how social learning may be influenced by misperceptions about prevailing norms. This is a critical omission because, as Smuts (1995) identified, the propagation of ideologies of male dominance likely played, and continues to play, an important and unique role in shaping the resolution of gendered conflict worldwide (Lawson et al., 2023). Drawing inspiration from both the social norms and cultural evolution literature, here we consider how perceptions about the gender role ideology of others may influence trajectories of cultural change. Specifically, we consider the notion, now popular among social norm researchers (see below), that men tend to overestimate peer support for inequitable gender norms, making them resistant to cultural change.

The idea that peer beliefs and behaviours are susceptible to misperception, hereafter ‘norm misperception’, has long been associated with the social norms approach to behaviour change (Berkowitz et al., 2022; Bursztyn & Yang, 2022; Dempsey et al., 2018). For example, numerous studies dating back to the 1980s have established that university students overestimate peer alcohol and drug use (reviewed in Perkins, 2014). Norm misperception has since been documented for a diverse range of topics, such as public support for tackling climate change or for flexible work and paternity leave (Geiger & Swim, 2016; Miyajima & Yamaguchi, 2017; Munsch et al., 2014). In general, these studies demonstrate that people overestimate the prevalence of problematic or socially undesirable behaviours and underestimate the prevalence of healthy or socially desirable behaviours. Norm misperception has also been described in terms of generalised biases of ‘pluralistic ignorance’ and ‘false consensus’, wherein, respectively, those in the majority (e.g. those who consume low or moderate amounts of alcohol) falsely perceive that they are in the minority, while those in the minority (e.g. those that consume large amounts of alcohol) falsely perceive that they are in the majority (Berkowitz et al., 2022; Dempsey et al., 2018; Perkins, 2014).

Recent research has focused on gender role ideology, particularly among men (Berkowitz et al., 2022). The direction of reported misperceptions is strikingly consistent. For example, Kilmartin et al. (2008) report that American college students overestimate the extent to which their peers hold sexist values. Berry-Cabán et al. (2020) report that American soldiers overestimate the prevalence of problematic beliefs associated with sexual aggression. Sobotka (2022) reports that, in an American online survey, men tend to believe that most men are more sexist than themselves (logically implying an overestimation of peer sexism). Extending the cross-cultural reach of this literature, Barnett (2023) documents a tendency for both genders, but especially men, to overestimate peer support for gender inequality in Morocco, while Bursztyn et al. (2020) demonstrate that men overestimate peer opposition to women's labour market participation in Saudi Arabia. In a follow-up study, this tendency to overestimate lack of peer support for women's basic rights was generalised to both men and women (although it was most pronounced among men) across 60 countries using national attitudinal surveys (Bursztyn et al., 2023). Several studies also present evidence that once men's perceptions are ‘recalibrated’ with ostensibly more accurate information provided by researchers gender norms change in meaningful ways (Berkowitz et al., 2022). For example, in Bursztyn et al. (2020) participants informed that they had overestimated peer opposition for female labour market participation were more likely to, in contrast to a control group, later report that their wife had applied and interviewed for a job in a follow-up survey.

### 1.2. Norm misperception or researcher measurement error?

The studies reviewed above suggest that the overestimation of peer support for gender inequity is widespread. Combined with a tendency to conform, norm misperception may therefore ultimately serve to reinforce inequitable gender norms, making them resistant to change (see also Berkowitz et al., 2022). However, the supporting literature behind this conjecture has important limitations. In particular, researchers routinely rely on conventional self-report measures to establish the allegedly true prevalence of beliefs and behaviours under study. Yet self-report measures, especially on sensitive topics like gender role ideology, are vulnerable to social desirability bias (Krumpal, 2013). As such, participants may feign a lack of support for inequitable gender norms (or falsely imply support for women's empowerment) to be viewed favourably by researchers. If they do, the common finding that participants overestimate presumed peer support for inequitable norms could represent nothing more than researcher measurement error. To take a parallel example, if individuals downplay their alcohol consumption to give more socially desirable responses, then they may in fact be relatively accurate when they state that others consume more alcohol than estimated by researchers.

This limitation has been recognised by proponents of the social norms approach. However, from our reading of the literature, the consensus appears to be that this is not a major cause for concern. There is some supportive evidence for this position. For example, in reviewing research on alcohol consumption, Perkins (2014) notes that key findings appear largely consistent even when using anonymous reporting methods or breath analyser studies to estimate consumption. In Bursztyn et al.'s (2023) cross-national study of support for female market participation, participants give very similar responses when asked to report on the extent to which others ‘would say that they agree’ and ‘would truly agree’ with statements, which the authors argue indicates that social desirability bias is unlikely to have impacted their results (see also discussion in Bursztyn & Yang, 2022). Nonetheless, it is our impression that this issue has received less attention than it deserves. For example, in Berkowitz et al.'s (2022) otherwise very extensive review of the social norms approach to violence perpetrated by men and boys, the potential limitations of self-report data and implications of social desirability are not discussed.

The present study provides an opportunity to address this shortcoming. We previously provided evidence that men misrepresent and exaggerate, often quite substantially, personal support for women's empowerment in self-report data (Lawson, Schaffnit, Kilgallen, et al., 2021). In a semi-representative survey of a single Tanzanian community (see below), we asked men to self-report their agreement with 20 statements. Independently, we then asked their wives to report what they thought their husbands believed about the same statements. The results were striking. For example, while 26% of men self-reported that they agreed that ‘A man is justified in hitting his wife if she disagrees with him’, 61% of their wives stated that their husbands would agree. Similarly, self-report data indicate that 10% of men agreed that ‘Education is more important for boys than girls’, while 22% of their wives said their husbands would agree. Differences in self- and wife-reported attitudes were most pronounced among men expressing the greatest support for women's empowerment (i.e. those declaring the lowest support for inequitable norms). While this discrepancy could hypothetically result from wives misperceiving or intentionally misreporting the attitudes of their husbands, we argue that it is more parsimoniously explained by social desirability bias affecting men's self-reported attitudes, such that wife-reported measures offer a more accurate, albeit still imperfect, measure of men's true beliefs. This interpretation is supported by previous studies demonstrating men's tendency to declare attitudes that appear favourable to interviewers, such as in reporting more support for gender equality when interviewed by a woman rather than a man (e.g. Charles, 2019), and a lack of equivalent incentives for women to misreport their husbands’ attitudes (for further discussion of this interpretation see Lawson, Schaffnit, Kilgallen, et al., 2021).

Here, we build on this research by using data we simultaneously collected on men's perceptions of the beliefs of other men in their community. These data allow us to provide a novel test of the hypothesis that men overestimate peer support for inequitable norms. By considering if observed patterns are robust to using our alternative wife-reported measure of men's beliefs, we can also test the counter hypothesis that apparent norm misperception is an artefact of researcher mismeasurement error. Using both measures, we furthermore assess the associated hypothesis that norm misperception acts to reinforce individual level support for gender inequity. If this is true, the more a man overestimates peer support for inequitable norms, the less we expect him to personally support women's empowerment. In other words, we anticipate that men who are less aware of emerging gender equitable attitudes in the community to be more likely to conform to relatively patriarchal values.

### 1.3. Why might peer gender role ideology be misperceived?

Beyond the general concepts of pluralistic ignorance and false consensus, theory development concerning the mechanisms driving norm misperception and, by extension, which types of individuals will be most likely to misperceive local norms, is limited and ununified (Berkowitz et al., 2022; Dempsey et al., 2018). Here, we suggest three non-mutually exclusive factors are particularly relevant to the misperception of gender role ideology.

First, individuals may falsely infer private beliefs from publicly observed behaviours. This could happen, for example, when those who deviate from perceived norms intentionally obscure their beliefs from others, motivated either by a generalised tendency to conform and avoid costs of norm violation (Henrich & Boyd, 1998; Morgan & Laland, 2012), or more specifically, because support for women's empowerment contradicts traditional ideals of masculine strength. Supporting this idea, relatively ‘feminist’ men have been observed to suffer various penalties, such as social exclusion or prejudice in employment decisions (Brough et al., 2016; Heilman & Wallen, 2010; Kågesten et al., 2016; Sideris, 2004). There is also evidence that men are aware of, and weigh the risks of, appearing weak when supporting women in front of other men, such as in decisions to intervene when observing sexual violence (Carlson, 2008). This dynamic of men intentionally misrepresenting their views to conform to masculine ideals or stereotypes can itself be considered a parallel form of social desirability bias, and may account for why men rather than women appear especially prone to overestimating peer support for gender inequity.

A second possibility is that in communities undergoing cultural change assumptions about the beliefs of others may be based on outdated or ‘lagged’ information (Miyajima & Yamaguchi, 2017; Vandello & Cohen, 2004). Since gender norms often become more equitable with market integration and economic development, reliance on outdated information could therefore contribute to norm misperception in communities that are undergoing rapid urbanisation. Furthermore, older individuals are more likely to be subject to lags in their beliefs by simple virtue of having more exposure to outdated information. In comparison, younger individuals only have relatively recent social observations to guide their impression of peer beliefs and may also have more exposure to relatively new social information via recent education or greater social media use. Some studies of social learning have also found that individuals tend to engage in more horizontal social learning early in life (e.g. Demps et al., 2012; Hewlett et al., 2011; Morgan et al., 2015), which could cause younger individuals to have a greater awareness of changing gender roles and the emergence of relatively equalitarian beliefs.

Finally, norm misperception may be influenced by wider patterns of access to and ability to evaluate the credibility of social information, which itself may vary in its accuracy. In this regard, public misunderstanding of the state of global affairs has been linked to the purposeful dissemination of fake news, along with biases in media coverage towards information that engages readers’ attention, i.e. ‘clickbait’ (de Oliveira & Albuquerque, 2021). Rosling et al. (2019), for example, argue that the tendency for news media to focus on negative events leads people to overestimate the global prevalence of poverty (see also Acerbi, 2019; Brand et al., 2019 for discussion of hypothesised content-biases for negative information). Similarly, the actions of global health agencies, who often focus on extreme negative scenarios in order to gain donor and public support, may cause confusion at local levels about the prevalence of problematic behaviours, such as intimate partner violence (IPV) or early marriage (e.g. Schaffnit et al., 2021). In general, we might expect that educational attainment will improve an individual's exposure to reliable social information and lead to a greater ability to discern the credibility of social information from indirect sources, such as news media or external agencies.

In summary, our study addresses the following hypotheses. First, we hypothesise that men will overestimate peer support for gender inequity, and that evidence for this pattern of norm misperception will be robust to potential researcher measurement error, as revealed by contrasting results based on self- and wife-reported measures of men's attitudes. Second, we hypothesise that men who overestimate peer support for gender inequity the most will be the least supportive of women's empowerment. This result would be consistent with men seeking to conform to perceived norms. Third and fourth, we hypothesise that older men and less educated men will be most prone to norm misperception based on the rationale outlined above. All data are drawn from a semi-representative sample of young men resident in an urbanising community in Mwanza, northern Tanzania. Situating our study in this context broadens the diversity of samples addressed in prior literature. Furthermore, by focusing on a community known to be undergoing rapid cultural changes in response to urbanisation, we anticipate that any tendency for norm misperception will be relatively pronounced.

## 2. Methods

### 2.1. Study context

Data collection was carried out within the Magu Health and Demographic Surveillance System (HDSS), managed by the Tanzanian National Institute of Medical Research (NIMR) and located in northwestern Tanzania, approximately 20 km east of Mwanza city. The HDSS comprises over 35,000 residents and has been monitored since 1994 (Kishamawe et al., 2015). The study community primarily identifies as Sukuma and is characterised by social norms favouring gender inequality, while also showing signs of changing gender roles associated with recent urbanisation (Kilgallen et al., 2022). Women increasingly work outside the home, but domestic chores and childcare are still considered to be primarily the obligation of women and girls (Hedges et al., 2018; Schaffnit, Hassan, et al., 2019). Educational attainment has increased in recent years, with girls’ education increasingly prioritised among families, sometimes catching up or exceeding boys’ education (Hedges et al., 2018). Intimate partner violence is commonplace; in a 2019 survey approximately one-third of women reported experiencing IPV in the last year, and almost two-thirds of women reported that their husband condones IPV (Kilgallen et al., 2022). Self-report data indicate preferential childcare of infant sons over daughters by fathers, but not mothers (Hassan et al., 2019). Fostering with close kin is common, especially for daughters; around a quarter of children between 7 and 19 years live apart from both parents (Hedges et al., 2019; Urassa et al., 1997).

Marriage frequently occurs during female adolescence and spousal age gaps can be large (Lawson, Schaffnit, Hassan, et al., 2021; Schaffnit, Urassa, et al., 2019), reinforcing gendered power inequalities. Most women report autonomy in navigating the marriage market, sometimes even marrying against parental wishes (i.e. eloping), but bridewealth is practised and is likely to constrain choice for some (Schaffnit, Urassa, et al., 2019; Baraka et al., 2022). Polygyny is permitted but has become less common in more urban areas (Lawson, Schaffnit, Hassan, et al., 2021). Virginity is not a pre-requisite for marriage, and childbearing before marriage is relatively common (Boerma et al., 2002), as is transactional sex (Wamoyi et al., 2019). Divorce may be initiated by either partner, and is typically followed quickly by remarriage, at least for individuals of childbearing age (Boerma et al., 2002). For a wider discussion of changing gender norms in Tanzania, see Badstue et al. (2020).

### 2.2. Sampling

Data collection took place between June and August 2019. The 2018 HDSS was used as a sampling frame to identify married men aged between 25 and 40 years and with at least one living child. Occasionally, men provided ages that did not match the HDSS. If the man's self-reported age at the time of survey was within 5 years of our selection criteria, he was included in our final sample (i.e. men aged 20–45 years are included). After locating eligible participants, men were interviewed at their residences if they were present. If the man could not be located, we asked present family or neighbouring community members for his whereabouts and attempted to visit him if he was within accessible range. For a subsample of participants, a similar procedure was applied to sampling men's wives. Sampling in this way proved challenging because of participants’ frequent daily movements between homes and work locations, as well as migrations for seasonal work. As a result, our sample is biased towards men (and their wives) most likely to be found at or near their homes at the time of the survey (for full details on sampling, see Lawson, Schaffnit, Kilgallen, et al., 2021).

Surveys were conducted separately with men and their wives in Kiswahili by Tanzanian researchers of the same gender, and all responses were recorded on tablets using Open Data Kit (Hartung et al., 2010). While in more rural settings the use of electronic tablets may feasibly introduce bias by emphasising that the interviewer is a community outsider, we doubt this mode of data collection is particularly influential in this urbanising context where exposure to similar technology such as smartphones and computers is common. Surveys were originally designed in English, and then translated to Kiswahili. Translations were then double-checked during team training (most of our team are at least partially bilingual), and piloting in the community, before being finalised. Comprehension of survey questions was high, which is to be expected since most young men in this community have attended formal schooling (although the majority only to the primary level; see Results). In rare cases where participants hesitated for long periods or expressed confusion, questions were rephased and/or examples offered. Regular team meetings across the project ensured that these techniques were employed as consistently as possible. Before all surveys began, participants were read a consent form and, if they agreed to be surveyed, were interviewed. Surveys always took place in a private space, away from other community members.

### 2.3. Measuring support for inequitable gender norms

**Figure 1.**
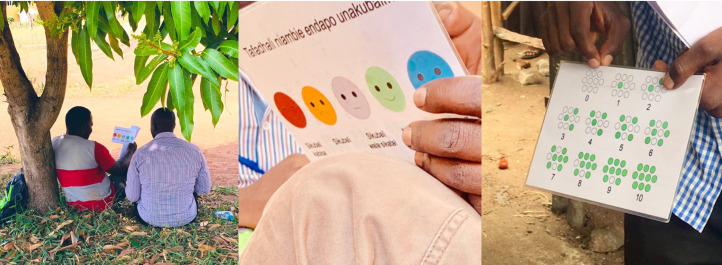
A participant survey. All surveys were conducted in private settings (left panel). Men were first asked to report their relative agreement or disagreement with 20 statements relating to gender roles. A visual aid of possible responses (strongly agree, agree, neither agree nor disagree, disagree, strongly disagree) was used to reinforce the use of all available response options (middle panel). After declaring their own attitudes, participants were then asked how many out of 10 hypothetical peers they estimate would agree or strongly agree with the same 20 statements. Once again, a visual aid was used to reinforce the use of all available response options (right panel).

Support for inequitable gender norms was assessed using a 20-item questionnaire. Questions were initially drawn from the ‘Women's Empowerment – Multidimensional Evaluation of Agency, Social Capital & Relations’ instrument (CARE USA, 2020), but modified to ensure they were contextually appropriate (see Lawson, Schaffnit, Kilgallen, et al., 2021). The topics include authority in decision-making, IPV, responsibility in childcare and family planning, women's economic independence, involvement in community affairs, sex biases in parental care and the viability of women's roles beyond marriage and motherhood. For each item, men were asked about their level of agreement with relevant statements from strongly agree, agree, neither agree nor disagree, disagree or strongly disagree (Figure 1). These data form our direct self-reported assessment of men's beliefs. The same procedure was then repeated with the wives of each participant, who were asked to report their husband's anticipated level of agreement with each item. As noted in our introduction, this indirect ‘wife-reported’ measure of men's beliefs generally indicates substantially greater support for inequitable gender norms. This is consistent with men mispresenting their true beliefs in self-report surveys owing to social desirability bias.

A composite measure was also constructed, using alternatively self-reported or wife-reported beliefs, to summarise men's overall support for inequitable gender norms. For consistency with our prior research (Lawson, Schaffnit, Kilgallen, et al., 2021), this measure is coded so that a greater value indicates greater support for women's empowerment (i.e. lower support for inequitable gender norms). It weighs responses to each question equally across all items and ranges from 0 to 100, with 100 indicating the strongest possible support and a score of 0 indicating the weakest possible support for women's empowerment. For those who responded ‘don't know’ or refused to answer up to five statements, a multiplication factor was applied to the total so that the maximum possible score equals 100. In contrast, participants with five or more ‘don't know’ or ‘refuse’ responses are excluded from analyses requiring this measure (see Lawson, Schaffnit, Kilgallen, et al., 2021 for details).

### 2.4. Measuring perceptions of peer beliefs

To assess the accuracy of men's perceptions of peer beliefs we first constructed measurements of community-wide support for inequitable gender norms using both self-reported and wife-reported beliefs. To do this we first formed a binary measure for each response, coded as ‘1’ if participants reported that they would agree or strongly agree with the items and ‘0’ otherwise. Given that greater agreement indicates greater support for gender inequity in some items but the opposite in others, we reverse code variables as necessary so that greater agreement uniformly shows *more* support for inequitable gender norms across all items. Community beliefs were then calculated as the percentage that support inequitable norms for each item (e.g. 8% of men self-reported that they agreed that it is better to have more sons than daughters in a family, while 17% of men's wives reported their husbands would agree with this statement). For each item, participants who refused to answer specific items or claimed that they did not know about their husband's beliefs (one to six male participants in self-report cases and 11–39 female participants in wife-report cases depending on the item) were excluded from the analysis of community-wide support.

Men's estimation of peer beliefs was then assessed by asking men, after they had reported their own beliefs, “*We are doing this survey with married men in [the study community] who are 25 through 40 years old. We would like to know what you think these other men believe about men and women's relationships. I am now going to read you some statements. If we were to speak to 10 married men between the ages of 25–40 in your community, how many of them do you think would agree with each of these statements?*” (Figure 1). Following this procedure for all 20 items, we derived the proportion of men estimated to support inequitable gender norms for each item. Comparing this data with our measures of men's actual beliefs (using both direct and indirect measures; see above), we then calculated an ‘overestimation score’ and ‘inaccuracy score’ as two alternative measures of men's overall ability to estimate peer attitudes.

The overestimation score represents the tendency of men to systematically overestimate or underestimate peer support for gender inequity. It is calculated as the mean difference between the estimated and the ‘actual’ proportion supporting inequitable norms for each of the 20 items based on either men's self or wife-reported beliefs (with relevant items reverse coded as described above as necessary). Differences were coded such that a higher positive value indicates a greater overestimation of peer support for inequitable gender norms, zero indicates a perfectly accurate estimate, and a more negative value means a greater underestimation. In contrast, the inaccuracy score is calculated as the mean of the absolute differences between men's estimated and actual peer support for inequitable gender norms for each of the 20 items. For this measure, the directionality of the difference between each estimate and the actual beliefs is not considered, with any difference coded as a positive value. A larger inaccuracy score, therefore, indicates a greater deviation from the actual beliefs, regardless of the tendency to overestimate or underestimate. Eight male participants failed to estimate at least one item and were excluded, leaving 581 participants in the analysis of men's perceptions of peer beliefs.

### 2.5. Analysis

We conducted all analyses in R. Accompanying code is included in the Supplementary Information. Our primary hypothesis is that men overestimate peer support for inequitable gender norms. To evaluate this hypothesis, we visualise the distribution of estimates for each item graphically and compare each distribution to our self-reported and wife-reported measures of men's beliefs (estimated with 95% confidence intervals) using one-sample Wilcoxon rank-sum tests. This test was selected because men's estimation responses were skewed (McCrum-Gardner, 2008). Tests are one-sided as we are specifically testing for evidence that men overestimate peer support for inequitable gender norms. To compare the magnitude of the differences between the distribution of estimates and self- and wife-reported measures of community beliefs, effect sizes were also calculated using the R Package ‘rcompanion’ (Mangiafico, 2023).

The remainder of our analysis concerns our overall scores for tendencies to overestimate and inaccurately perceive peer support for gender inequity. We first compare each of these scores as estimated by self or wife-reported measures using *t*-tests. Then to test whether men who overestimate peer support for inequitable gender norms will be the least supportive of women's empowerment, we run a simple linear regression predicting our summary measure of support for women's empowerment across all domains and visualise the associations with the overestimation and inaccuracy scores using scatter plots. To examine the hypotheses that men who are older and with less education will have greater overestimation and inaccuracy of peer support for inequitable gender norms, we use multiple linear regression. Analyses are presented using both direct (self-reported) and indirect (wife-reported) assessments of men's beliefs, allowing us to determine if patterns of norm misperception are influenced by the form of measurement.

### 2.6. Ethical approval

Ethical approval for this study was granted by UCSB's Office of Research (4-19-0247), the Tanzanian National Institute for Medical Research Lake Zone Institutional Review Board (MR/53/100/595) and the Tanzanian National Ethical Review Committee (NIMR/HQ/R.8a/Vol.IX/3104). Approval to carry out the study was also obtained at the community level following a presentation of the study objectives, requirements and projected outputs to community leadership.

## 3. Results

### 3.1. Study participants

A total of 590 men and 317 of their wives were surveyed. The mean age of the male participants was 34.7 years (standard deviation, SD = 4.7) and the mean age of their wives was 29.4 (SD = 5.3). While 3% of the men had failed to complete any education, the majority had completed either primary school only (67%) or had advanced to secondary school (24%). Men with post-secondary education (6%) were rare. Similarly, most women had attended either primary school only (67%) or advanced secondary school (21%), while 9% had received no formal education. Note that sampling of men's wives was restricted owing to budgetary constraints. See Lawson, Schaffnit, Kilgallen, et al. (2021) for additional descriptive data on the sample and confirmation that self-reported beliefs of men sampled alone did not differ from those whose wives also contributed data.

### 3.2. Do men overestimate peer support for inequitable gender norms?

Table 1 shows the percentage of peers estimated to support inequitable gender norms and the corresponding percentage supporting inequitable gender norms, as measured alternatively by self- and wife-reported beliefs. When taking the conventional approach of using men's self-reported beliefs to measure community beliefs, we find striking evidence of a generalised tendency for men to overestimate peer support for inequitable norms. For 18 out of 20 statements, men estimated that significantly more men supported inequitable norms than their self-reported beliefs imply. Accordingly, the central tendency (i.e. mean and medians) of men's estimates are typically greater than, and outside of the 95% confidence intervals of, the actual percentage of men who claimed support for inequitable gender norms across almost all measures. The magnitude of discrepancies varies widely, with effect sizes ranging from close to zero to 0.67. The mean effect size across all 20 statements is 0.43, which is generally regarded as a moderate to large effect size. The largest effect size corresponds to the statement “*It is better to have more sons than daughters in a family*”. On average, men estimated that around 40% of their peers would agree with this statement. In contrast, only 8% of surveyed men self-reported that they agreed with this statement.

**Table 1.** Perceived and actual support for inequitable gender norms as measured by self- and wife-reported beliefs

Statements	Percentage estimated to support inequitable gender norms		Percentage who support inequitable gender norms (95% CIs)		Wilcoxon rank-sum test effect size	
	Mean	Median	Self-reported	Wife-reported	Self-reported	Wife-reported
1. It is better to have more sons than daughters in a family	38	40	8 (6–11)*	17 (13–22)*	0.67	0.53
2. It is important for women to earn their own money	37	40	22 (18–25)*	16 (12–21)*	0.4	0.49
3. A husband and wife should decide together equally about when to have children	25	20	5 (4–8)*	7 (4–10)*	0.46	0.46
4. Only men should be allowed to manage a business	37	30	7 (5–9)*	15 (11–20)*	0.62	0.45
5. Only men should be allowed to own land	40	40	9 (7–12)*	22 (17–27)*	0.65	0.44
6. Education is more important for boys than girls	38	30	10 (8–13)*	22 (18–27)*	0.56	0.38
7. Women should express their opinions at community meetings	18	0	2 (1–4)*	4 (2–7)*	0.38	0.38
8. Women should be welcome at community meetings	16	0	3 (2–5)*	4 (2–7)*	0.33	0.33
9. It is the mother's responsibility alone to take care of the children	35	30	11 (9–14)*	22 (18–27)*	0.51	0.31
10. A woman can live a successful life even if she does not marry a man	31	20	21 (18–24)*	23 (18–28)*	0.21	0.21
11. If a woman wants to avoid being pregnant, it is her responsibility alone to prevent the pregnancy	39	40	15 (12–18)*	34 (28–40)*	0.58	0.14
12. A woman should tolerate being beaten by her husband to keep her family together	43	40	29 (25–33)*	42 (36–48)	0.35	0
13. A man should have the final say about decisions in his home	69	80	63 (59–67)*	73 (67–78)	0.21	0
14. A man is justified in hitting his wife if she refuses to have sex with him	34	30	5 (4,8)*	35 (29–40)	0.66	−0.01
15. It is important for girls/women to be educated	10	0	1 (0–2)	2 (1–5)	−0.01	−0.01
16. Only when a woman has a child is she a real woman	49	50	25 (21–29)*	52 (46–58)	0.56	−0.14
17. A man is the one who decides when to have sex with his wife	50	50	30 (26–34)*	56 (50–62)	0.53	−0.21
18. A man is justified in hitting his wife if she argues with him	51	50	26 (22–30)*	61 (55–66)	0.55	−0.32
19. A woman should be free to divorce (or leave) her husband even if he does not wish	53	50	51 (47–55)	68 (63–74)	−0.02	−0.36
20. A wife should be able to prevent her husband from taking another wife	58	60	48 (44–52)*	76 (70–81)	0.32	−0.50

Support for inequitable gender norms is indicated by agreeing with or not agreeing with statements opposing and favouring women's empowerment respectively.

**Figure 2.**
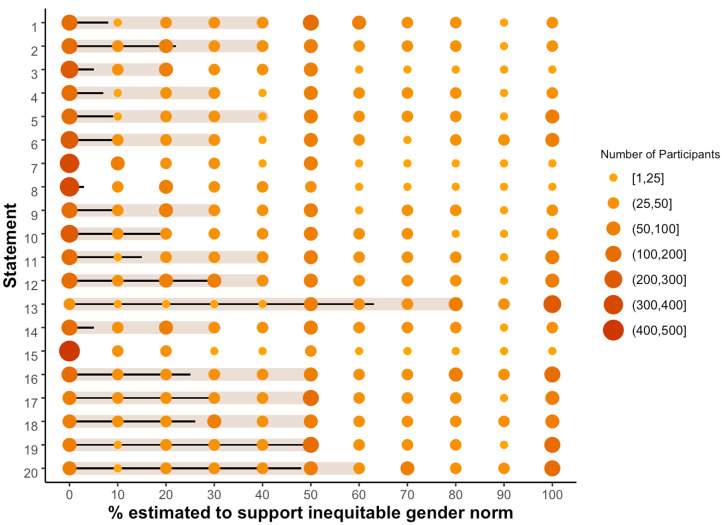
A graphical depiction of the distribution of estimates of peer support for inequitable gender norms compared to actual support as based on self-reported beliefs. Using self-reported measures of men's beliefs implies that men overestimate peer support for inequitable gender norms, often quite substantially. According to this measure, for 18/20 statements, men estimated that significantly more of their peers would support inequitable gender norms than actually do. Bubbles represent the distribution of men's estimates, with the size of the bubble indicating the number of participants making each possible estimate and the thick shaded bar indicating the median estimate. The thin black line represents the actual percentage of men who supported inequitable gender norms for each statement based on self-reported beliefs. Statements are numbered to match Table 1. Support for inequitable gender norms is indicated by agreeing with or not agreeing with statements opposing and favouring women's empowerment respectively. Figure S1 also presents this data as conventional histograms for each statement. See Table 1 for statistical test results and confidence intervals.

**Figure 3.**
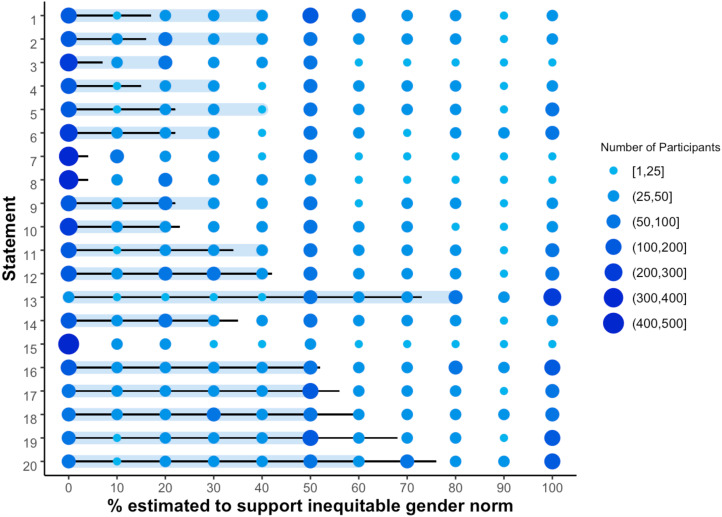
A graphical depiction of the distribution of estimates of peer support for inequitable gender norms compared with actual support as based on wife-reported beliefs. Using wife-reported measures of men's beliefs also implies that men overestimate peer support for inequitable gender norms. However, this tendency is reduced in magnitude and scope. According to this alternative measure, which we suggest more accurately measures men's beliefs, for 11/20 statements presented, men estimated that significantly more of their peers would support inequitable gender norms than actually do. Bubbles represent the distribution of men's estimates, with the size of the bubble indicating the number of participants making each possible estimate and the thick shaded bar indicating the median estimate. The thin black line represents the actual percentage of men who supported inequitable gender norms for each statement based on wife-reported beliefs. Statements are numbered to match Table 1. Support for inequitable gender norms is indicated by agreeing with or not agreeing with statements opposing and favouring women's empowerment respectively. Figure S2 also presents this data as conventional histograms for each statement. See Table 1 for statistical test results and confidence intervals.

One-sample, one-sided Wilcoxon rank-sum tests are used to test if men overestimated the percentage of men supporting inequitable gender norms (* *p* < 0.001); see Table S1 for full test statistics. Statements are arranged in descending order by effect size when using wife-reported beliefs to measure community norms. Statement numbering matches Figures 2 and 3.

The picture changes when using our novel indirect measure to assess men's beliefs. According to wife-reported measures, in 11 out of 20 statements men overestimated the percentage of their peers supporting inequitable gender norms. The mean effect size across all 20 statements is 0.13, which is generally regarded as a small effect size. Furthermore, for six statements (i.e. those in the bottom rows of Table 1) the direction of men's misperception appears to be flipped. For example, the median estimate for the percentage of men agreeing that “*A man is justified in hitting his wife if she argues with him*” was 50%. In contrast, 26% self-reported agreeing with this statement (implying overestimation of peer support), but 61% of men's wives reported that their husbands would agree (implying underestimation of peer support). Overall, using wife-reported measures of men's beliefs confirms a general tendency to overestimate peer support for inequitable gender norms. However, using this arguably more accurate measure of men's true beliefs also implies that this pattern is not universal. Rather the degree to which men overestimate peer support for inequitable norms is more modest in scope and magnitude, and, in some cases, men may even underestimate the degree to which their peers support gender inequity.

Figures 2 and 3 graphically present the distribution of men's estimations of local community beliefs for all 20 statements, contrasted with the actual percentage self- and wife-reported to support inequitable norms respectively. Comparing these figures illustrates the general pattern we observe: the overestimation of peer support for inequitable gender norms is less pronounced when using wife-reported measures of men's beliefs. They also illustrate that men's estimates are generally not normally distributed, with estimated percentages of men supporting inequitable gender norms clustering around 0, 50 and 100%. Conventional histograms for all 20 statements are also presented in the Supplementary Information (Figures S1 and S2).

**Figure 4.**
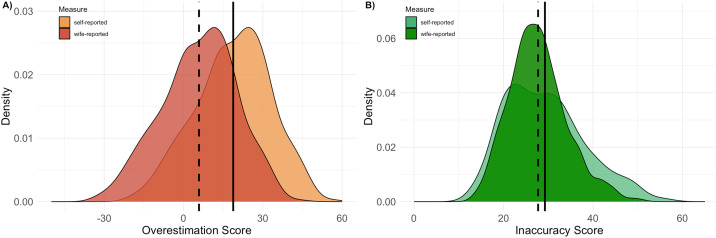
The distribution of men's (a) overestimation and (b) inaccuracy scores, as calculated based on men's self- and wife-reported beliefs. Apparent tendencies to both overestimate peer support for inequitable gender norms and make inaccurate estimates in either direction are significantly lower when using wife as opposed to self-reported measures of men's beliefs. Vertical lines display mean values for self-reported (solid line) and wife-reported measures (dashed lined). See text for means, standard deviations and statistical tests for differences between self- and wife-reported measure based scores.

Figure 4 presents men's overall overestimation and inaccuracy scores, summarising tendencies for norm misperception across all items combined. As expected, the overestimation score is lower when community-level support for inequitable norms is measured using wife-reported beliefs (mean = 5.8, SD = 14.2) as opposed to self-reported beliefs (mean = 18.8, SD = 14.2; *t*(1160) = 15.54, *p* < 0.001). These scores can be interpreted as men estimating, on average, between 6 and 19% more of their peers supporting inequitable norms than actually do. Men's inaccuracy scores, which measure deviations between the estimated norm and actual norm regardless of directionality, were more similar, but nevertheless also significantly lower when using wife-reported beliefs (mean = 27.7, SD = 6.4) as opposed to self-reported beliefs (mean = 29.3, SD = 8.9) to measure of community-level support for inequitable norms (*t*(1058) = 3.36, *p* < 0.001). These scores can be interpreted as men, on average, estimating a level of peer support for inequitable norms 28–29% away from the actual level of support. These results confirm that using our indirect, and likely more accurate, wife-reported measure of men's beliefs reduces implied norm misperception, and particularly the directionality of apparent errors in perception.

### 3.3. Is norm misperception associated with men's support for women's empowerment?

**Figure 5.**
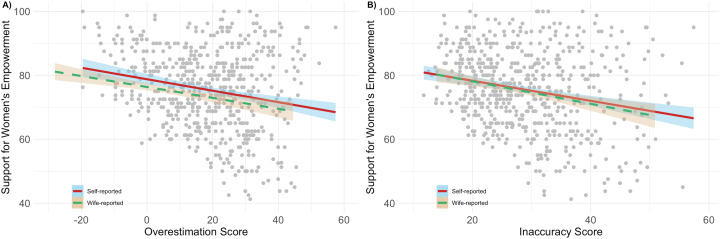
Scatterplots showing the association of (a) overestimation and (b) inaccuracy scores with men's self-reported support for women's empowerment. Men who overestimate and make inaccurate estimates of peer support for inequitable gender norms to a larger degree tend to self-report lower support for women's empowerment. These relationships hold when using both self-report and wife-report based measures of the overestimation and inaccuracy score. See text for supporting statistics.

Now that we have confirmed that men tend to overestimate peer support for inequitable gender norms, we can assess whether this tendency is associated with their own support for women's empowerment. Figure 5 suggests that this is the case. The more that men overestimate peer support for inequitable gender norms the lower their self-reported support for women's empowerment (Figure 5a, Pearson's *r* = −0.20, *p* < 0.001; Pearson's *r* = −0.19, *p* < 0.001, using self- and wife-reported based measures of the overestimation score respectively). Likewise, the more inaccurate a man's perception of peer beliefs is, the lower his self-reported support for women's empowerment (Figure 5b, Pearson's *r* = −0.22, *p* < 0.001; Pearson's *r* = −0.18, *p* < 0.001, using self- and wife-reported based measures of the inaccuracy score respectively). These patterns are consistent with the notion that misperception of local norms, and specifically an overestimation of peer support for relatively inequitable beliefs about gender, stifles positive change in gender role ideology.

### 3.4. What types of men are most likely to misperceive local gender norms?

Finally, we examine how men's characteristics are associated with patterns of norm misperception (Table 2). Confirming our predictions, older men overestimate peer support for inequitable norms to a greater degree than younger men. Compared with men under 30 years, men over 40 years overestimate peer support for inequitable norms more (*B* = 4.44, 95% CI = [0.48, 8.40], *p* < 0.05). This pattern of results is equivalent when using either self- or wife-reported beliefs as our measurement of community norms because the alternative calculation only shifts the distribution of scores to lower values (see Figure 4, and differing intercept in Table 2). The overall degree of inaccuracy, however, was not significantly associated with men's age.

**Table 2.** Multiple linear regressions predicting men's overestimation and inaccuracy scores by age and education

		Overestimation score				Inaccuracy score			
		Self-reported		Wife-reported		Self-reported		Wife-reported	
		*B*	95% CI	*B*	95% CI	*B*	95% CI	*B*	95% CI
Intercept		23.28***	15.83, 30.70	10.32**	2.88, 17.77	35.65***	31.01, 40.29	33.93***	30.57, 37.29
Age (reference, <30 years)	30–35 years	2.16	−1.20, 5.53	2.16	−1.20, 5.53	0.73	−1.37, 2.83	−0.03	−1.55, 1.49
	36–40 years	3.04**†**	−0.55, 6.63	3.04**†**	−0.55, 6.63	0.72	−1.51, 2.96	−0.31	−1.93, 1.31
	40+ years	4.44*	0.48, 8.40	4.44*	0.48, 8.40	1.53	−0.94, 4.00	−0.61	−2.40, 1.18
Educational attainment (reference, no education)	Primary	−7.02**†**	−14.34, 0.31	−7.02**†**	−14.34, 0.31	−6.96**	−11.53, −2.40	−5.90***	−9.21, −2.60
	Secondary	−8.42*	−15.94, −0.90	−8.42*	−15.94, −0.90	−8.46***	−13.15, −3.77	−6.58***	−9.97, −3.18
	Higher education	−2.58	−11.20, 6.04	−2.58	−11.20, 6.04	−6.80*	−12.17, −1.43	−7.28***	−11.17, −3.39
*N*		581		581		581		581	
*r*^2^		0.03		0.02		0.02		0.03	

*** *p* < 0.001; ** *p* < 0.01; * *p* < 0.05; † *p* < 0.1

We also find that educational attainment was associated with both the overestimation and inaccuracy score, such that, as predicted, greater education is associated with lower overestimation and inaccuracy. For example, compared with men who have not had any education, men who attended secondary school had an approximately 8-point reduction in their overestimation score (*B*_self-reported_ = −8.42, 95% CI = [−15.94, −0.90]; *p* < 0.05) and an 8-point reduction in their inaccuracy score (*B*_self-reported_ = −8.46, 95% CI = [−13.15, −3.77]; *p* < 0.001). These patterns are approximately consistent regardless of whether we use self or wife-reported measures of men's beliefs in our calculation of the overestimation score. Model fit statistics indicate that these relationships only explain a small fraction of the total variance in patterns of norm misperception.

## 4. Discussion

### 4.1. Errors in norm perception and measurement

We add to a now ample body of research on norm misperception concluding that men overestimate the extent to which their peers support inequitable gender norms (Berkowitz et al., 2022; Bursztyn et al., 2023). On the one hand, our findings could be interpreted as supporting the assertion that this tendency is cross-culturally pervasive and generalisable to multiple domains of gender role ideology. While past research has focused on North American settings and primarily on gendered violence or women's labour market participation, here we present supporting data from an urbanising African context and utilise a much broader range of measures, encompassing statements relating to economic and reproductive autonomy, support for IPV, marital relations, parental care, education and community involvement. Furthermore, we demonstrate that men who overestimated peer support for gender inequity to the largest degree also reported the lowest levels of support for women's empowerment. This suggests that (mis)perception of peer beliefs exerts an important influence on individual gender role ideology, helping to explain why inequitable norms can be resistant to change (see also Casey et al., 2020; Fabiano et al., 2003; Thébaud & Pedulla, 2016; Witte & Mulla, 2012).

On the other hand, we also conclude that both participants and researchers are error prone when estimating community norms, calling into question the validity of findings of past studies relying on conventional self-reported measures of men's beliefs. We previously demonstrated that men self-report lower support for inequitable gender norms than their wives state they do (Lawson, Schaffnit, Kilgallen, et al., 2021). Here, we further demonstrate that when using wife-reported measures of local norms, men's apparent tendency to overestimate peer support for gender inequity is lessened in magnitude (smaller effect sizes) and scope (applying to half as many statements as compared to analyses based on self-reported measures). Given that wives may also misreport or misperceive the beliefs of their husbands to some extent, we consider these results strongly suggestive rather than fully diagnostic evidence that social desirability bias creates an illusion of norm misperception. Nevertheless, our results cast a shadow of doubt over previous literature confidently declaring that norm misperception of gender role ideology is a robust and widespread phenomenon (Berkowitz et al., 2022; Bursztyn et al., 2020, 2023; Bursztyn & Yang, 2022).

This conclusion reinforces the need for more sophisticated indirect measures of sensitive beliefs (see also Cloward, 2014; Gibson et al., 2018; Lindstrom et al., 2010; Nillesen et al., 2021). This issue is particularly pertinent because social desirability bias could logically apply not only to self-reported beliefs but also to reported estimates of the attitudes of one's peers. The direction of potential bias in the latter case is particularly difficult to forecast because individuals may balance a desire to portray their community favourably against a desire to portray others less favourably so that they themselves appear as *relatively* desirable in contrast to their peers. Moreover, these considerations signify a need for caution before rolling out social norm interventions poised to ‘correct’ apparent norm misperception (Bursztyn et al., 2020; Kilmartin et al., 2008). Our results imply that such interventions have previously unrecognised potential to misinform community members about prevailing beliefs. While it might be countered that this is of little consequence if the result is positive social change, deviation from prevailing norms may also carry meaningful costs to individuals, such as well-documented ‘backlash effects’ whereby advances in women's empowerment are met with increased IPV as men try to retain the status quo (Kilgallen et al., 2022).

Assuming that wife-reported data present a relatively accurate measure of men's true beliefs (for discussion see Lawson, Schaffnit, Kilgallen, et al., 2021), the emergent question from our study becomes: why did men tend to overestimate peer support for inequitable gender norms for some statements and not others? (Table 1, Figure 3). Most notably, and troublesome for advocates of the social norms approach to gendered violence (Berkowitz et al., 2022), evidence for norm misperception with regard to our three statements on IPV disappears when we switch to using wife-reported beliefs to measure local norms. Not coincidentally, these uniquely sensitive statements appear particularly vulnerable to social desirability bias, with men's wives reporting that their husbands support IPV much more than their husbands self-report. One possible explanation is that men may be relatively good at estimating peer beliefs which are stable across time and space, which in this case may apply to topics such as the acceptability of IPV and male authority (e.g. “*A man should have the final say about decisions in his home*”; “*A man is the one who decides when to have sex with his wife*”). In contrast, men may be less good at estimating beliefs that have recently or are currently undergoing social change, because such change will logically lead to vulnerability to outdated social information. Supporting this speculation, evidence for norm misperception appears most convincing for statements relating to women's social and economic autonomy (e.g. “*It is important for women to earn their own money*”; “*Only men should be allowed to manage a business*”), corresponding to recent changes in gender roles in the community, such as increases in women's education and labour market participation (Kilgallen et al., 2022). Whatever the case, the important conclusion here is that misperception of peer support for inequitable gender norms is unlikely to be universally applicable to all dimensions of gender role ideology.

### 4.2. The causes of norm misperception

A further advancement of the current study is our exploration of individual variation in patterns of norm misperception, providing novel clues to the mechanisms at play. Consistent with our predictions, relatively young men overestimated peer support for inequitable norms to a lesser degree. We suggest that this is reflective of older people, by virtue of their greater life experience, accumulating more exposure to now outdated social information. As this urbanising study community is moving towards more equitable gender norms (Kilgallen et al., 2022), older men may consequently falsely believe that others hold relatively inequitable beliefs and be less aware of emerging support for women's empowerment in the community. In addition, younger people may be more actively engaged in social learning. Several studies have found transitions in learning strategies under different life stages in humans: from strong vertical social learning in childhood, to more oblique and horizontal social learning in adolescence and greater individual learning in adulthood (Demps et al., 2012; Hewlett et al., 2011). However, we also note the age range of our study is narrow, leaving uncertainty about how the patterns observed here extrapolate to both older and younger ages than we considered.

Relatively educated men were more accurate in their judgments of peer beliefs. Again, this may be explained by several non-mutually exclusive mechanisms. First, more educated men may have acquired advanced skills in social perception, improving their capacity to decipher honest signals of other men's beliefs. Second, education may also lead to social changes, e.g. in employment and movement around the community, such that highly educated men gain a better appreciation of the diversity of viewpoints held by their community. Third, the pattern we observe here may reflect differences in reference groups, rather than actual differences in the accuracy of norm perception. We have previously established that well-educated men are more supportive of women's empowerment (Lawson, Schaffnit, Kilgallen, et al., 2021; see also Charles, 2019; Kyoore & Sulemana, 2019). As such, if highly educated men primarily socialise with other highly educated men, their assessments of community-wide attitudes may be based on a biased sample of the community, counteracting alternative mechanisms that otherwise lead men to overestimate peer support for inequitable norms.

A more mundane explanation is that more educated men were more able to comprehend our survey question asking them to assess peer beliefs (see also Hruschka et al., 2018 for a broader discussion of mismatch between survey instruments and cultural differences in skills, motivations and modes of social interaction). During data collection, we observed that some men initially found the question difficult to follow, probably reflecting unfamiliarity with this style of questioning (while participants have taken part in surveys in the community as part of the ongoing HDSS, questions about the attitudes of others are novel). However, in cases of participant confusion, questions did not continue until participants expressed that they comprehended. Furthermore, such confusion cannot obviously explain asymmetric errors in norm misperception, i.e. the tendency to overestimate, rather than simply be inaccurate.

Age- and education-related mechanisms presumably create a capacity for norm misperception in both genders. In addition, and as outlined in the introduction, ‘social performance’ of locally ideal masculine stereotypes (see also Badstue et al., 2020; Dery et al., 2022) may play a specific and important role in shaping men's perceptions about one another's beliefs. Although we have no means of testing this idea in the present study, qualitative research in Tanzanian settings reports that private support for gender equality is often masked in community interactions, with individuals who support women's empowerment privately nevertheless strategically upholding a ‘gender norms façade’ so as to imply conformity to socially desirable beliefs (Badstue et al., 2020; Galiè & Farnworth, 2019). Anecdotally, we also observed that men frequently joked about male authority in ways seemingly at odds with their personally expressed values (noted in Lawson, Schaffnit, Kilgallen, et al., 2021). If men portray allegiance to inequitable gender norms to one another via their social interactions in this way, this could contribute to an overestimation of peer support for inequitable beliefs.

Our study raises further questions about the nature of norm (mis)perception that lie beyond the scope of the presented analysis. Men's estimates of how many of their peers would agree/disagree with each statement tended to cluster around 0, 100 and 50% (Figures 2 and 3). There are at least two potential explanations for this phenomenon. The first is that it reflects a shortcoming of our measurement tool. Participants were directly encouraged to answer across the full range of alternatives via both verbal prompts and a visual aid (Figure 1). However, it is feasible that a lack of interest or commitment to our survey led to imprecise estimates being shared. While this is a serious concern that could lead us to falsely infer inaccuracy in men's estimations, it cannot obviously account for the systematic directionality in norm misperception we observe. The second possibility is that clumping in men's estimates reflects how social cognition truly works in this context, such that people make estimates of peer beliefs as broader mental constructs that lack the precision of our survey tool. Supporting this notion, during data collection participants typically made statements such as or ‘almost no people will believe this’ or ‘around half will agree’, before being encouraged to select a more precise estimate on our survey tool. While we cannot differentiate these alternative explanations, we emphasise that this question only came to light because we examined the distribution of men's estimates rather than just measures of central tendency. We encourage future research to follow this approach and experiment with alternative methods to assess norm (mis)perception. It would also be instructive to consider if the patterns we observe apply to women's propensity for norm misperception, which has so far been neglected by applied studies more typically targeting change in men's beliefs and behaviour (Berkowitz et al., 2022).

### 4.3. Implications for the social learning of gender roles

Evolutionary and social learning orientated perspectives on gender have sometimes been pitted against each other (e.g. Wiederman & Allgeier, 1993). Recognising that our propensity for social learning itself evolved as an adaptive mechanism (Kendal et al., 2018; Morgan et al., 2012) eschews this dichotomy, and it is our sense that few researchers today, in or outside of the evolutionary human sciences, would doubt that gender role ideology, and by extension gender differences in behaviour, are in large part culturally acquired. Yet, despite this consensus, and a long-standing interest in ‘gender role socialisation’ across the social sciences (John et al., 2017; Stockard, 2006), the social learning of gender role ideology remains somewhat opaque, and has received only limited attention by researchers adopting a cultural evolution framework (Lawson et al., 2023). In recent theoretical work, Cross et al. (2023) argue that gender roles are best understood as products of domain-general social learning biases, such as well-established tendencies to conform with the majority which may reinforce differentiation in gendered social networks, or tendencies to imitate self-similar individuals such as those of the same sex. In small-scale ancestral human societies, it is argued that these mechanisms largely guide individuals towards locally appropriate (i.e. adaptive) behaviour, while also being capable of creating relatively arbitrary gendered patterns, particularly in more evolutionary novel environments.

Our contribution should serve a reminder that as we continue to construct and test evolutionary accounts of social learning, in any domain, we cannot assume that individuals always have access to accurate information on the beliefs and behaviours of others. To advance our understanding of the implications of inaccuracies in social information, we advocate that future research would do well to gather data, not just quantifying norm misperception, but also on how the beliefs and behaviours of peers, and other influential members of social groups, are rendered visible through daily social interactions. Studies of this type may be best grounded in observational methodologies, which may inform us of variations in ‘social diet’ (Nettle et al., 2012), along with qualitative investigations into how men and women conceptualise and discuss social information among themselves. Gender role ideology is particularly fascinating to consider in this regard, because some domains, such as the involvement of women and men in community affairs, are likely to be more visible than, for example, what happens between wives and husbands at home.

## 5. Conclusion

We document a tendency to overestimate peer support for gender inequity in a Tanzanian community where gender roles are shifting with urbanisation. These findings add to a growing literature proposing that norm misperception is a pervasive phenomenon generally, and specifically with respect to gender role ideology. However, in contrast to many prior studies on the topic, we also present evidence that measurement error may lead this tendency to be exaggerated by researchers; apparent norm misperception is attenuated when using a novel indirect measure of men's beliefs. Future research on the social learning of gender role ideology would benefit from continued methodological refinement in the measurement of both community norms and individual perceptions of those norms. Further theoretical and empirical developments are also needed to address the causes of norm misperception and the applicability of these mechanisms to alternative dimensions of gender ideology. Collaboration between evolutionary social scientists studying cultural evolution and proponents of the social norms approach to behaviour change will be instrumental in meeting these goals.

## Supporting information

Lawson et al. supplementary material 1 Lawson et al. supplementary material

Lawson et al. supplementary material 2 Lawson et al. supplementary material

## Data Availability

n/a, see above.

## Supplementary material

To view supplementary material for this article, please visit https://doi.org/10.1017/ehs.2024.6

## Research transparency and reproducibility

The data that support the findings in this study are available from the corresponding author and the National Institute for Medical Research, Tanzania, on request, providing that necessary steps are taken to ensure participant anonymity is maintained. Raw data are not suitable for being made publicly available owing to their sensitive nature and the possibility of participant identification.
