# Supplementary material for: Misperception of peer beliefs reinforces inequitable gender norms among Tanzanian men
Source: Evol Hum Sci. 2024 Feb 21;6:e17. doi: 10.1017/ehs.2024.6 (PMC10988154; doi:10.1017/ehs.2024.6)
Supplement: Lawson et al. supplementary material 2 — Lawson et al. supplementary material [file S2513843X24000069sup002.docx]

Supplementary Information for Lawson et al. “Misperception of peer beliefs reinforces inequitable gender norms among Tanzanian men.”

**Table S1** Perceived and actual support for gender inequitable gender norms as measured by self and wife-reported beliefs, and results of one-sample, one-sided Wilcoxon rank-sum tests used to test if men overestimated the % of men supporting inequitable gender norms for each statement.

|  | % estimated to support inequitable gender norms | | % who support inequitable gender norms | | p-value for Wilcoxon rank sum test | | V-value for Wilcoxon rank sum test | | r-value for Wilcoxon rank sum test | |
| --- | --- | --- | --- | --- | --- | --- | --- | --- | --- | --- |
| Statements | Mean | Median | self-reported (95% CIs) | wife-reported (95% CIs) | self-reported | wife-reported | self-reported | wife-reported | self-reported | wife-reported |
| *It is better to have more sons than daughters in a family* | 37.7 | 40 | 8.26 (6.15,10.8) | 17.29 (13.15,22.1) | 0 | 0 | 149975 | 135997 | 0.67 | 0.53 |
| *It is important for women to earn their own money* | 37.4 | 40 | 21.51 (18.24,25.08) | 16.23 (12.25,20.88) | 0 | 0 | 123818 | 132396 | 0.4 | 0.49 |
| *A husband and wife should decide together equally about when to have children* | 25.3 | 20 | 5.38 (3.69,7.55) | 6.9 (4.26,10.45) | 0 | 0 | 129057 | 129057 | 0.46 | 0.46 |
| *Only men should be allowed to manage a business* | 37 | 30 | 7.06 (5.11,9.45) | 15.18 (11.33,19.73) | 0 | 0 | 144992 | 128116 | 0.62 | 0.45 |
| *Only men should be allowed to own land* | 40.2 | 40 | 9.47 (7.21,12.14) | 21.69 (17.13,26.84) | 0 | 0 | 147426 | 127815 | 0.65 | 0.44 |
| *Education is more important for boys than girls* | 37.5 | 30 | 9.98 (7.67,12.71) | 22.15 (17.56,27.3) | 0 | 0 | 138630 | 121083 | 0.56 | 0.38 |
| *Women should express their opinions at community meetings* | 17.6 | 0 | 2.41 (1.32,4.01) | 3.95 (2.06,6.79) | 0 | 0 | 120555 | 120555 | 0.38 | 0.38 |
| *Women should be welcome at community meetings* | 15.8 | 0 | 3.44 (2.12,5.27) | 3.96 (2.06,6.82) | 0 | 0 | 116421 | 116421 | 0.33 | 0.33 |
| *It is the mother’s responsibility alone to take care of the children* | 34.8 | 30 | 11.38 (8.91,14.25) | 22.04 (17.51,27.13) | 0 | 0 | 134521 | 114781 | 0.51 | 0.31 |
| *A woman can live a successful life even if she does not marry a man* | 30.5 | 20 | 20.9 (17.66,24.44) | 22.84 (18.13,28.12) | 0 | 0 | 104880 | 104880 | 0.21 | 0.21 |
| *If a woman wants to avoid being pregnant, it is her responsibility alone to prevent the pregnancy* | 38.8 | 40 | 14.66 (11.88,17.8) | 33.79 (28.39,39.52) | 0 | 0 | 140675 | 97835 | 0.58 | 0.14 |
| *A woman should tolerate being beaten by her husband to keep her family together* | 43 | 40 | 29.09 (25.42,32.97) | 42.21 (36.45,48.14) | 0 | 0.49 | 118665 | 84595 | 0.35 | 0 |
| *A man should have the final say about decisions in his home* | 68.5 | 80 | 62.99 (58.92,66.93) | 72.79 (67.42,77.7) | 0 | 0.48 | 104525 | 84694 | 0.21 | 0 |
| *A man is justified in hitting his wife if she refuses to have sex with him* | 33.8 | 30 | 5.34 (3.66,7.5) | 34.55 (29.19,40.22) | 0 | 0.63 | 148092 | 83176 | 0.66 | -0.01 |
| *It is important for girls/women to be educated* | 10.2 | 0 | 0.52 (0.11,1.5) | 2.33 (0.94,4.73) | 0.64 | 0.64 | 83166 | 83166 | -0.01 | -0.01 |
| *Only when a woman has a child is she a real woman* | 48.6 | 50 | 24.83 (21.36,28.55) | 52.1 (46.14,58.01) | 0 | 1 | 139205 | 70416 | 0.56 | -0.14 |
| *A man is the one who decides when to have sex with his wife* | 49.9 | 50 | 29.83 (26.13,33.73) | 56.23 (50.38,61.95) | 0 | 1 | 135669 | 63914 | 0.53 | -0.21 |
| *A man is justified in hitting his wife if she argues with him* | 50.6 | 50 | 25.82 (22.3,29.58) | 60.74 (54.94,66.32) | 0 | 1 | 138336 | 53589 | 0.55 | -0.32 |
| *A woman should be free to divorce (or leave) her husband even if he does not wish* | 52.9 | 50 | 51.21 (47.06,55.35) | 68.35 (62.52,73.77) | 0.68 | 1 | 82679 | 49701 | -0.02 | -0.36 |
| *A wife should be able to prevent her husband from taking another wife* | 57.7 | 60 | 47.76 (43.63,51.91) | 75.63 (70.15,80.55) | 0 | 1 | 115418 | 36105 | 0.32 | -0.5 |
| r-value indicates the effect size of the one-sample Wilcoxon rank-sum test: 0.1 - 0.3 = small effect; 0.30 - 0.5 = moderate effect; >0.5 =large effect, the same standard applies to the negative values | | | | | | | | | | |

**Figure S1** Self-reported measures of men’s beliefs imply that men overestimate peer support for inequitable gender norms, often quite substantially. According to this measure, for 18/20 statements presented men estimated that significantly more of their peers would support inequitable gender norms than actually do. See Table S1 for statistical test results and confidence intervals.

**Figure S2** Wife-reported measures of men’s beliefs also imply that men overestimate peer support for inequitable gender norms. However, this tendency is reduced in magnitude and scope. According to this alternative measure, which we suggest more accurately measures men’s beliefs, for 11/20 statements presented, men estimated that significantly more of their peers would support inequitable gender norms than men actually do. See Table S1 for statistical test results and confidence intervals.
